# 2593. Epidemiology and Clinical Burden of Invasive Pneumococcal Disease in Older Adults in the City of São Paulo, Brazil

**DOI:** 10.1093/ofid/ofad500.2208

**Published:** 2023-11-27

**Authors:** Licieri Figueiredo, Eitan N Berezin, Thiago J A Silva, Maisa Carla Kairalla, Daniel Jarovski, Milene Fernandes, Cícera P Marcelino, Paula M Batista, Marina D N Paula, Thais Moreira

**Affiliations:** MSD Brazil, São Paulo, Sao Paulo, Brazil; Santa Casa de São Paulo, São Paulo, Sao Paulo, Brazil; Hospital das Clínicas da Faculdade de Medicina da Universidade de São Paulo, São Paulo, Sao Paulo, Brazil; Universidade Federal de São Paulo, São Paulo, Sao Paulo, Brazil; Santa Casa de São Paulo, São Paulo, Sao Paulo, Brazil; CTI Clinical Trial and Consulting Services, São Paulo, Sao Paulo, Brazil; MSD LATAM, São Paulo, Sao Paulo, Brazil; MSD LATAM, São Paulo, Sao Paulo, Brazil; MSD Brazil, São Paulo, Sao Paulo, Brazil; MSD Brazil, São Paulo, Sao Paulo, Brazil

## Abstract

**Background:**

The Brazilian National Immunization Program currently provides pneumococcal vaccination for children and high-risk adults. However, the burden of invasive pneumococcal disease (IPD) remains poorly described, especially among older adults. We evaluated the incidence, demographics, clinical characteristics, and outcomes among older adults hospitalized with IPD in São Paulo (SP), the largest city in Brazil.

**Methods:**

Retrospective chart review of adults ≥ 60y hospitalized with IPD at three tertiary teaching hospitals from January 2016 to December 2018. Incidence was determined, only for people living in SP, using as numerator the number of IPD cases at study hospitals and as denominator the proportion of admissions at study hospitals considering the whole SP public system among people ≥ 60 living in SP. Demographic and clinical data were described for all patients with information.

**Results:**

Ninety-four patients were included: 98.9% had ≥ 1 selected risk factor (most frequent: cancer, 68.2%), and 90% had unknown vaccination status (Table 1). The 3-year incidence was 121.8 per 100,000 people (60-74y: 117.6 and ≥ 75y: 132.6), while annual incidences per 100,000 people were 47.5 in 2016, 40.1 in 2017, and 34.2 in 2018. Most patients had community-onset disease (89.4%) and the main presentation was bacteremic pneumonia (68.1%), followed by meningitis (10.6%). The median hospitalization length was 10 days, 53.4% of patients had ICU stay (median duration of 7 days). About 22.2% had antimicrobial resistance to more than one class. During hospitalization, 85.1% of patients had ≥ 1 complication (Figure 1), 32.3% developed septic shock, 63.8% required supplemental oxygen, and 36.2% required mechanical ventilation (median: 5 days). In-hospital mortality was 42.6% and, among survivors (n=54), 20.4% reported sequelae (mostly chronic respiratory failure).

**Figure:**
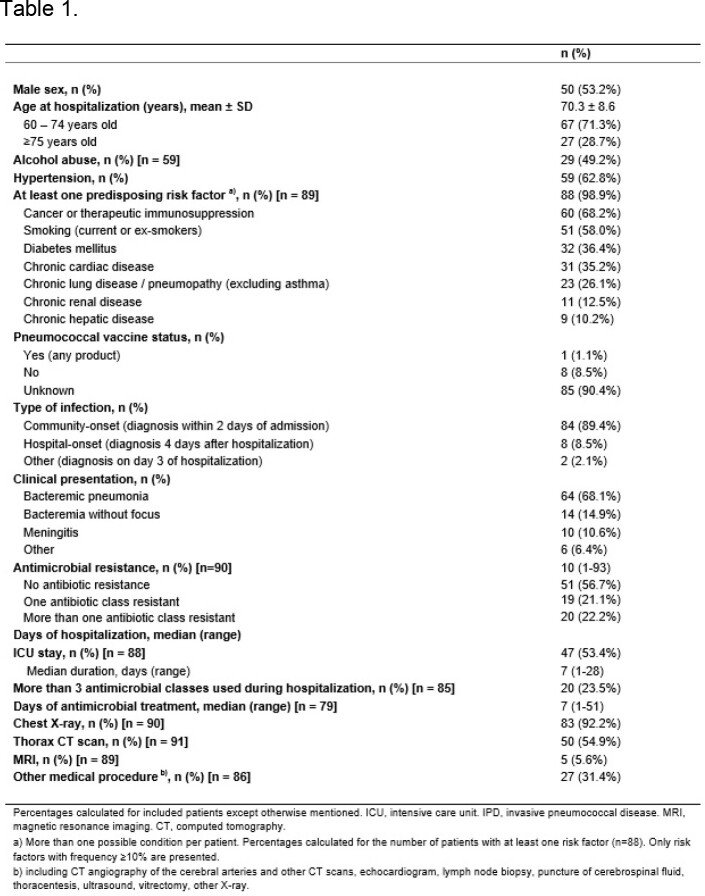

Demographic and clinical characteristics of older adults with invasive pneumococcal disease, São Paulo city, 2016-2018 (n=94)

**Figure:**
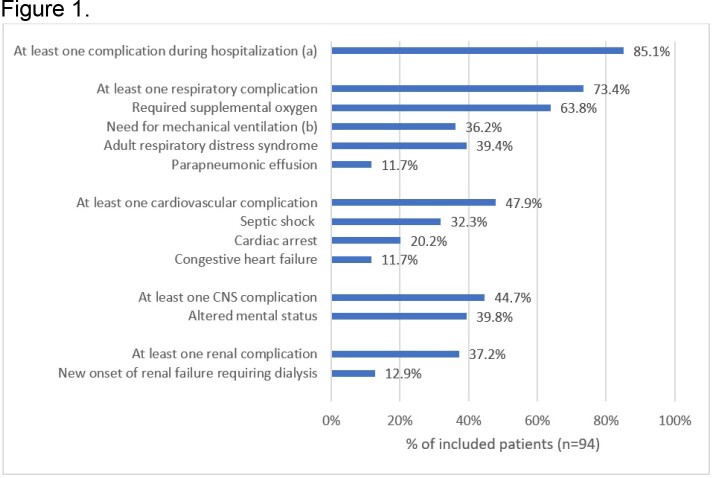

Complications during hospitalization due to of invasive pneumococcal disease Note: Percentages calculated for all included patients. CNS, central nervous system. (a) More than one complication possible per patient. Only complications with frequency ≥10% are described. (b) Median (range) duration of mechanical ventilation support: 5 (1-28) days.

**Conclusion:**

This study showed a high burden of IPD in older adults, with higher incidence than reported for other countries such as the USA (26 /100,000 people ≥ 65 y in 2018) or Chile (12.1 /100,000 people ≥ 65y in 2018). The important rates of morbidity and mortality, and a considerable burden on healthcare resources support the importance to expand and improve pneumococcal prevention for older population in Brazil.

**Disclosures:**

**Licieri Figueiredo, MD**, MSD Brazil: This is an employee of the MSD company leading the study **Cícera P. Marcelino, n/a**, MSD LATAM: This is an employee of the MSD company responsible for the management and operation of the study **Paula M. Batista, n/a**, MSD LATAM: This is an employee of the MSD company responsible for the management and operation of the study **Marina D N Paula, n/a**, MSD Brazil: This is an employee of the MSD company who is the leader of the therapeutic area of the study **Thais Moreira, MD, MSc**, MSD LATAM: This is an employee of the MSD company that provides scientific support for the study regionally

